# Encapsulation of *Salmonella* phage SL01 in alginate/carrageenan microcapsules as a delivery system and its application *in vitro*

**DOI:** 10.3389/fmicb.2022.906103

**Published:** 2022-08-04

**Authors:** Yuqiao Zhou, Dingting Xu, Haijie Yu, Jianzhong Han, Weilin Liu, Daofeng Qu

**Affiliations:** ^1^School of Food Science and Biotechnology, Zhejiang Gongshang University, Hangzhou, China; ^2^The Second Affiliated Hospital, School of Medicine, Zhejiang University, Hangzhou, China; ^3^Jiaxing Vocational Technical College, Jiaxing, China

**Keywords:** phages, alginate, κ-carrageenan, composite microcapsules, encapsulation

## Abstract

Phages can be used successfully to treat pathogenic bacteria including zoonotic pathogens that colonize the intestines of animals and humans. However, low pH and digestive enzyme activity under harsh gastric conditions affect phage viability, thereby reducing their effectiveness. In this study, alginate (ALG)/κ-carrageenan (CG) microcapsules were developed to encapsulate and release phage under simulated gastrointestinal conditions. The effects of ALG and CG concentrations on the encapsulation and loading efficiency of microcapsules, as well as the release behavior and antibacterial effects of microcapsules in simulating human intestinal pH and temperature, were investigated. Based on various indicators, when the concentration of ALG and CG were 2.0 and 0.3%, respectively, the obtained microcapsules have high encapsulation efficiency, strong protection, and high release efficiency in simulated intestinal fluid. This effect is attributed to the formation of a more tightly packed biopolymer network within the composite microcapsules based on the measurements of their microstructure properties. Bead-encapsulation is a promising, reliable, and cost-effective method for the functional delivery of phage targeting intestinal bacteria.

## Introduction

*Salmonella* infections remain a big burden for public health worldwide. The illness causes 93.8 million foodborne cases and 155,000 deaths annually in developing countries including many regions of the African and Asian continent (Eng et al., 2015). There are three main types of Salmonellosis: (1) localized intestinal infection (gastroenteritis), (2) systemic infection of otherwise healthy hosts (typhoid), and (3) systemic infection of immune-compromised hosts (Griffin and McSorley, 2011; Hurley et al., 2014). In comparison, gastroenteritis is caused mainly by *S. enterica* Serovar Typhimurium (*Salmonella Typhimurium*) and Serovar Enteritidis, which are common even in developed countries. The mortality caused by typhoid *Salmonella* strains can be up to 7% even when antibiotics are used (Eng et al., 2015).

With the increasingly frequent appearance and expansion of multidrug-resistant bacteria, there has been a re-evaluation of the therapeutic use of phages (Beke et al., 2016; Kakasis and Panitsa, 2019). Phages are highly specific to each species and even subspecies of bacteria they target, and thus are ideally suited to eliminate foodborne pathogens from food surfaces and the human intestinal tract without disturbing the native microbiome. Furthermore, lytic phages do not cause human allergies, nor do they change the structure, odor, or flavor of food products (Hagens and Offerhaus, 2008). These characteristics make phages of interest for use in veterinary and human medicine, as well as in the food industry. More recently, several phage products targeting *Salmonella*, *Shigella* spp., and *Escherichia coli O157:H7* have received “generally recognized as safe (GRAS)” status from the U.S. Food and Drug Administration (FDA), and these products are currently successfully utilized in the food industry to safeguard the food chain (Febvre et al., 2019).

However, phages, when taken orally, should actively reach the target region to regulate human gut health. After oral uptake, phages are exposed to digestive conditions such as high acidity and enzymes during the passage through the gastrointestinal tract and lose their activity. Among the different routes of phage administration, the oral route is likely to be the most appropriate for phage therapy in humans and animals (Zelasko et al., 2017). The advantages include relatively easy administration, potentially low immunogenicity, and greater patient comfort compared to other parenteral modes of administration (Zelasko et al., 2017). Alginate is considered a good system for phage encapsulation because of its ability to resist acidity and control and sustain the release of live products to the gut, such as probiotic bacteria and phages (Gbassi et al., 2009). Alginate (Alg) polysaccharide can be obtained naturally from bacteria and algae, which crosslinks to form a gel with calcium (Lee and Heo, 2000). Typically, alginate microcapsules are made using the extrusion-gel method because it is a fast, simple, and gentle process (Wawrzyñska and Kubies, 2018). In particular, it does not involve any heat treatment, which is important for biologically active proteins, which are normally inactive when heat-denatured.

However, studies have shown that microcapsules produced by using only alginate could not protect phages against high acidity so that alginate is used in combination with various polymers such as chitosan, pectin, mannitol, whey protein, and gelled milk (Samtlebe et al., 2016; Vinner and Malik, 2018; Śliwka et al., 2019). It was reported that alginate and κ-carrageenan (CG) formed spherical and uniformly shaped composite microcapsules containing a biopolymer network held together by calcium ions. Compared with pure alginate microcapsules, the thermostability of the biopolymer networks has been reported to be improved by the addition of CG (Mohamadnia et al., 2008). Furthermore, these polysaccharide-based microcapsules are biocompatible and degradable, functional, and non-toxic, so they have great potential for use in phage delivery systems. We hypothesized that similar composite microcapsules could be used to enhance the oral delivery of phages. However, to the best of our knowledge, this information has been rarely reported before.

Consequently, this study aimed to microencapsulate a lytic *Salmonella* phage SL01 and focused on the effect of ALG and CG concentration on the formation and properties of AC-phage microcapsules, including their encapsulation efficiency, loading efficiency, resistance to gastric environments, and the release under simulated gastrointestinal conditions. These results can provide application references for the development of antibacterial and therapeutic agents based on microencapsulated phage, which has a huge application market.

## Materials and methods

### Phage and its host strain *Salmonella*

Phage SL01 and *Salmonella Typhimurium* LT_2_ (ATCC 700720) were grown in LB (Luria-Bertani) broth with shaking or on LB agar plates for 18 h at 37°C and were used to propagate and quantify phage SL01, which was obtained from previous isolation and stored in the laboratory. Amplified phages were isolated by centrifugation at 8000 × *g* for 10 min and filtration using 0.22 μm pore size disposable sterile syringe filters. The double-layer agar method was used to determine the titer of the phage stock. Aliquots from serial tenfold phage dilutions (100 μL) were used to infect early log-phase host cells (100 μL) at 0.3 OD_600_. After adsorption at room temperature, the phage-bacteria mixture was suspended in LB (soft agar, containing 0.6% agar) and poured on Petri plates containing LB (hard agar, containing 1.5% agar) for overnight incubation at 37°C.

### Characterization of phage

#### Stability of the phage at different pH values

The stability of the phage was assayed by adding 100 μL of phage lysate to 900 μL 0.2% (w/v) NaCl solutions whose were adjusted to pH 1–14 with 1 M HCl and were sterilized at 121°C for 15 min before use (Ma et al., 2008; Ergin et al., 2021). After incubation for 60 min at 37°C, the phage titers were determined with the double-agar layer method. The experiment was repeated three times.

#### Stability of the phage at different temperatures

The stability of phages over a range of temperatures was evaluated by incubating phage suspensions in SM buffer at 30, 40, 50, 60, 70, and 80°C for 15, 30, 45, and 60 min. The titers of phages were determined using the double-agar overlay plaque assays. The experiment was repeated three times.

#### Optimal multiplicity of infection

The multiplicity of infection (MOI) refers to the ratio of the number of phages to the number of host bacteria at the time of initial infection. The *S. Typhimurium* LT_2_ was grown in LB medium until the early-log-phase (1 × 10^8^ CFU/mL). According to a certain MOI (0.001, 0.01, 0.1, 1, 10, 100, 1000), the phage SL01 (500 μL) was mixed with an equal volume of the host bacteria in LB and then the mixture was incubated at 37°C with shaking (160 rpm) for 4 h. After centrifugation (8000 × *g*, 4°C) for 10 min, the supernatant was diluted and spotted onto a double-layer agar plate to determine the phage titer.

#### One-step growth curve

The latent period, rise period, and the lysis amount of phage are determined by a one-step growth curve. The phage SL01 (500 μL) was added to the LT_2_ culture at the infection multiple of 0.01. Then the mixture was incubated at 37°C for 20 min with shaking at 160 rpm and centrifuged (8000 × *g*, 4°C) for 2 min. Then the pellet was washed twice with LB broth and resuspended in 10 mL of LB. The suspension was immediately incubated at 37°C (160 rpm) after 100-fold dilution. Every 5 min, 100 μL of the sample was taken and centrifuged (8000 × *g*, 4°C) for 2 min. As the rise progresses, one can replace sampling of the original culture with 100 μL sampling of a 100-fold dilution (Hyman and Abedon, 2009). Then the supernatant was diluted and spotted onto a double-layer agar plate to determine the phage titer.

#### Transmission electron microscopy

Phage was examined using transmission electron microscopy (TEM) as previously described (Abdelsattar et al., 2019). Briefly, fixed phages on Pioloform grids using glutaraldehyde were negatively stained with 0.5% uranyl acetate. After drying, the specimens were examined using a JEM-1230 transmission electron microscope.

### Determination of apparent viscosity of encapsulated solution

Sodium alginate (Sinopharm Chemical Reagent, Shanghai, China) solutions were dissolved in distilled water to prepared 0.5–3.0% concentrations and sterilized at 121°C for 15 min. κ-Carrageenan (Sinopharm Chemical Reagent, Shanghai, China) was dissolved in distilled water to prepare 0.15–0.45% concentrations. The apparent viscosity of ALG and ALG-CG encapsulated solution was measured by rheometer. A plate with a diameter of 40 mm was selected, the shear rate was set at 200 s^–1^, and the distance between the plate and the test platform was 1.0 mm. After the temperature rose to 25°C, the apparent viscosity was measured.

### Preparation of alginate and AC microcapsules

An appropriate amount of phage was added to the ALG solution, stirred at room temperature, and then to stand overnight at 4°C for degassing. By the extrusion method, 22-G needle was used to drop phage-ALG solution into 150 mM CaCl_2_ crosslinking solution at 200 rpm. After standing for 30 min of crosslinking reaction, microcapsules were filtered through a sieve, and the microcapsules were cleaned repeatedly with a certain amount of sterile deionization water. The water on the surface of the microcapsules was sucked dry to obtain wet microspheres. Dried phage-ALG microspheres were obtained by drying them in a constant temperature oven to a constant weight.

An aqueous solution of CG was prepared at 60°C. After cooling to room temperature, it was mixed with a certain concentration of ALG solution, and then the phage was added to the ALG-CG mixture. ALG-CG (AC) microcapsules were prepared in the same way.

### Properties of composite capsule solutions

#### Encapsulation efficiency of phage

The phage titer in the microcapsules per gram wet weight was determined using a releasing solution, which contained 0.05 M sodium citrate, 0.2 M sodium hydrogen carbonate, and 0.05 M Tris-HCl (pH 7.5) (Samtlebe et al., 2016). The microencapsulation efficiency (EE, %) was calculated according to Equation


EE(%)=(P/0P)total×100

where EE represents the encapsulation efficiency, P_0_ (PFU/mL) represents the titer of phage released from the microcapsules, and P_total_ represents the titer of (PFU/mL) the phage in the microencapsulating mixture, which was determined using the double agar layer method.

#### Macroscopic morphology and microstructure of phage microcapsules

The surface water was absorbed from freshly prepared microcapsules, and the surface morphology of wet microcapsules was observed by a digital camera and microscope. The surface microstructure of dried microcapsules was observed by scanning electron microscope. Firstly, the particles are fixed on the sample table and then observed after spraying gold in a vacuum environment.

### Resistance of microencapsulated phage to gastric environment

The titer of phage was determined to evaluate their resistance to simulated gastric conditions (Kovacs-Nolan and Mine, 2005). In brief, dried microcapsules containing the phage were placed in simulated gastric fluids (SGF, pH 2.0, 0.32% pepsin, 0.2% NaCl) and then incubated at 37°C with 100 rpm shaking for 2 h. They were then separated by filtration and washed three times with sterile water. The microcapsules were then disrupted by placing them into disintegrating solutions consisting of a mixture of 0.2 M NaHCO_3_ and 0.06 M Na_3_C_6_H_5_O_7_⋅2H_2_O (pH 8.0). The titers of released phages were determined using the double-agar overlay plaque assays. They were separated by filtration and washed three times with deionized water.

### Release behavior of phage-loaded composite microcapsules

Initially, the microcapsules were added to 5 mL of simulated gastric fluid (SGF, pH 1.5, 0.2% NaCl) for 2 h and subsequently transferred to 5 mL of small intestinal fluid (SIF, pH7.0, 0.05 mol/L KH_2_PO_4_) for 5 h with 37°C and 100 rpm shaking. The swollen microcapsules were filtered from the gastrointestinal fluids at certain time intervals. The titers of released phages were determined using the double-agar overlay plaque assays. They were separated by filtration and washed three times with deionized water.

### Swelling degree of phage microcapsules digested *in vitro*

Dry microspheres of a certain quality were accurately weighed in 10 mL SGF preheated to 37°C and shaken for 2 h at 100 rpm. Then the particles were transferred to 10 mL SIF for further swelling for 5 h. During this period, the microcapsules were taken out at regular intervals to drain the surface moisture, and the quality of the microcapsules were determined to calculate the swelling degree (SD) of the microcapsules:


$$\text{SD}={\text{W}}_{\text{t}}/{\text{W}}_{0}$$

where SD represents the swelling degree of the microcapsules; W_t_ (g) represents the mass of the microcapsules after swelling; and W_0_ (g) represents the initial mass of microcapsules before swelling.

### Lytic activity assay

Encapsulated and non-encapsulated phages were tested for their lytic activity against LT_2_ by incubating each type of microcapsules in intestinal buffer at 37°C with agitation at 120 rpm. Exponentially growing cultures of strain LT_2_ were diluted with LB to an OD_600_ of 0.05. SL01 phage suspension to obtain a MOI of 0.01 and samples were collected after 2, 4, and 6 h of latent for analysis.

### Statistical analysis

The experimental data were analyzed using Graphpad Prism 8.1 for univariate analysis of variance, and the statistical differences between the two groups were analyzed using unpaired two-tailed *T*-tests. One-way ANOVA was used for statistical differences between multiple groups, and data results were expressed as mean ± SD. Different letters a–g indicate significant differences (*p* < 0.05).

## Results

### Characterization of phage SL01

**FIGURE 1:**
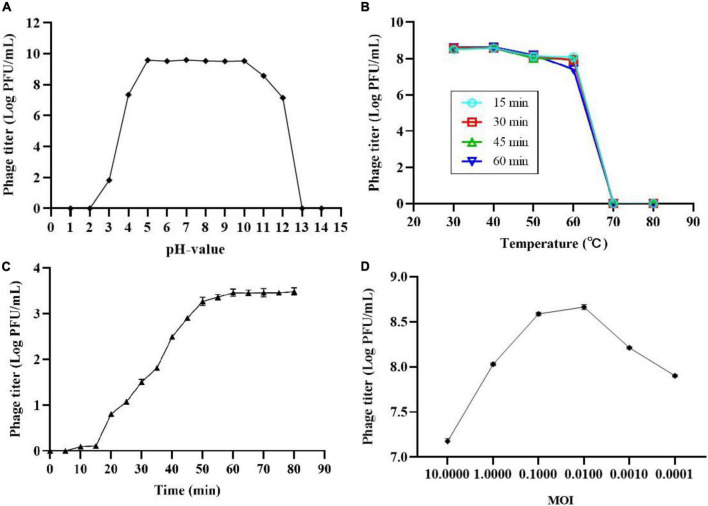
Biological characterization of phage SL01. **(A)** pH stability of SL01. **(B)** The optimal multiplicity of infection. **(C)** One-step growth curve of SL01. **(D)** The optimal multiplicity of infection.

The thermal and pH stability of phage SL01 were determined based on changes of phage count under various conditions. Results showed that phage was highly stable with environmental pH 4-10 (Figure 1A). However, it was completely abolished under strong acid or strong alkali (pH < 3.0 or pH > 12.0). The temperature stability test showed that the phage count did not show significant loss between 30 and 40°C (Figure 1B). At temperatures > 70°C, the phage count dramatically decreased.

One-step growth experiments were performed to assess the population kinetics of phage SL01 in the presence of *S. Typhimurium* (Figure 1C). The latent period of SL01 was about 15 min, which was shorter than that of many reported *Salmonella* phages (Huang et al., 2018; Soykut et al., 2019). Studies have shown that phages with short latent periods split more bacteria over a given period of time and are therefore better suited for biological control (Abedon, 1989). Subsequently, the phage titer increased dramatically in the next 45 min. The average burst size was calculated as approximately 37 PFU/CFU.

After mixing different titers of phage SL01 with host *Salmonella* and cultured for 4 h, the phage titers in the supernatant were determined by the double-layer plate method (Figure 1D). At MOI of 0.01, phage titer reached the highest.

### Apparent viscosity of microencapsulated encapsulated solution

**FIGURE 2:**
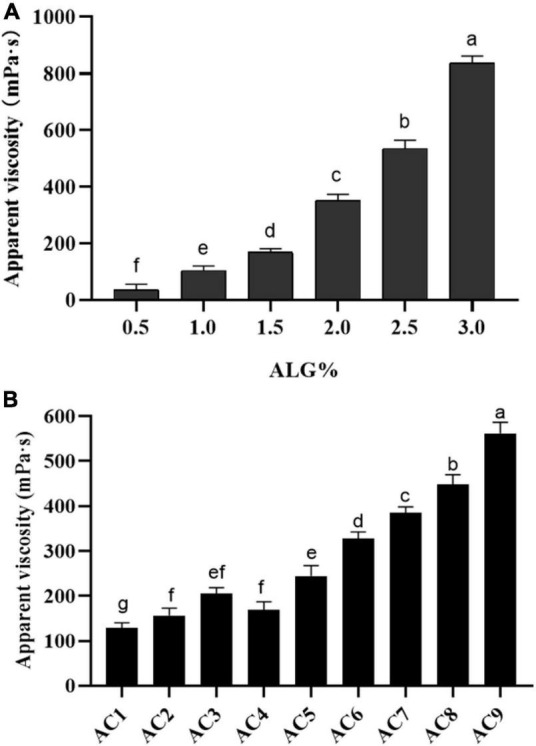
Apparent viscosity of ALG and ALG-CG encapsulation solutions. **(A)** Apparent viscosity of ALG with different concentrations. **(B)** Different proportions of ALG-CG, AC1-AC9 represents polysaccharide mixtures in different proportions. Different letters a–g indicate significant differences (*p* < 0.05).

The apparent viscosity of ALG and ALG-CG encapsulated solution was determined as shown in Figure 2, and the concentration of the encapsulated solution had a significant impact on the apparent viscosity (*p* < 0.05). When the concentration of ALG was 0.5%, the apparent viscosity is only 21.66 mPa⋅s, and the shape of the microspheres prepared was poor (Figure 2A). When the concentration of ALG was 2%, the apparent viscosity increases rapidly, which made the injection difficultly and was not conducive to the preparation of microcapsules (Figure 2A). As shown in Figure 2B, AC1-AC9 represents polysaccharide mixtures in different proportions, and with the increase of CG concentration, the apparent viscosity of ALG-CG solution increased significantly (*p* > 0.05). When ALG: CG was 2:0.45 (AC9), the apparent viscosity was more than 500 mPa⋅s, resulting in the blockage of the injection process and the morphology of the microspheres obtained was not uniform.

### Morphology and microstructure of microcapsules

**FIGURE 3:**
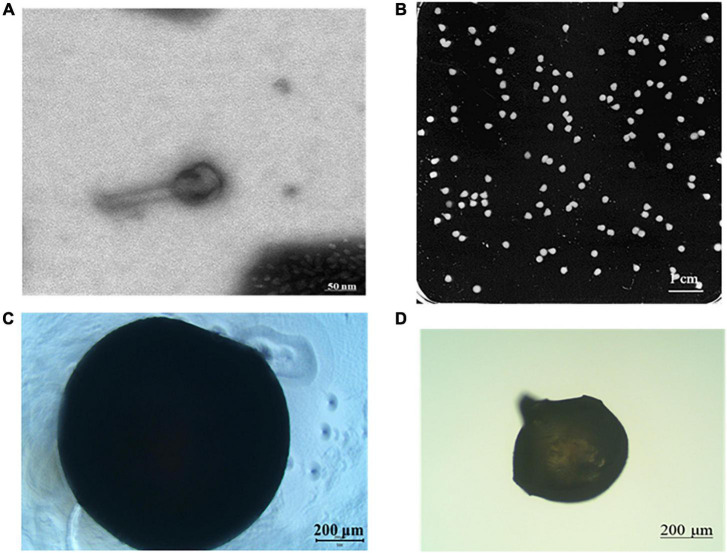
Morphology of phage and phage microcapsule. **(A)** Phage morphology; **(B)** microcapsules photographed by digital camera; **(C)** microscopic view of wet microcapsules; **(D)** microscopic view of dry microcapsules.

**FIGURE 4:**
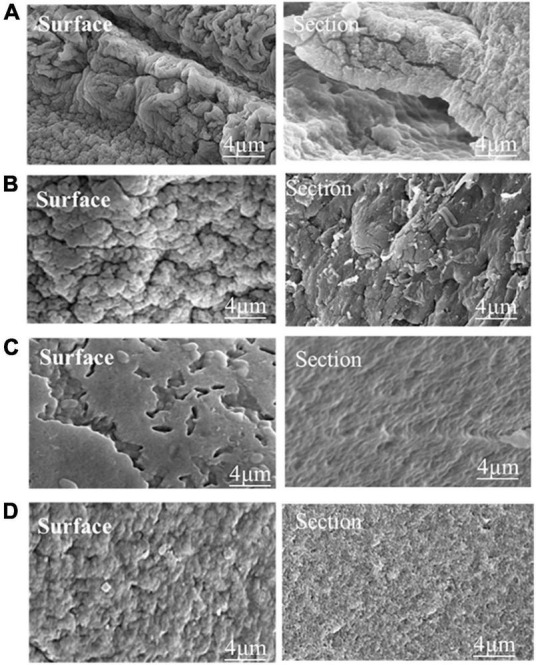
The microstructure of the dry microcapsules. **(A)** Pure ALG microcapsule. **(B)** ALG-phage microcapsule; **(C)** AC microcapsules; **(D)** AC-phage microcapsules.

Figure 3 shows the morphology of phage and phage microcapsules. The electron micrographs showed that SL01 had an icosahedral symmetry head (diameter of about 70 nm) and a tail (diameter of about 50 nm) (Figure 3A). Most of the microcapsules obtained by extrusion method were plump white microspheres (Figure 3B). The edge of the wet microcapsule was smooth and flat (Figure 3C), and the dry microcapsule could basically maintain the spherical granular shape (Figure 3D). The size of wet microspheres was in the range of 2–3 mm (Table 1). With the increase of the concentration of ALG solution, the particle size of microspheres also gradually increased. After adding CG, the encapsulation rate increased significantly, and there was a significant difference among different proportions. When ALG: CG was 2.0: 0.3, and 1.0: 0.45, the encapsulation rate was more than 95%. The surface and cross-section morphology of ALG microcapsule, ALG-phage microcapsule, AC microcapsule, and AC-phage microcapsule were compared under scanning electron microscope (Figure 4).

**TABLE 1:** Encapsulation rate and particle size of microcapsules.

	ALG (%)	CG (%)	Microencapsulation Efficiency (%)	Size of microcapsules (mm)
1	1.0	–	27.76 ± 1.22^h^	2.110 ± 0.291^d^
2	1.5	–	33.07 ± 0.98^g^	2.325 ± 0.354^c^
3	2.0	–	38.37 ± 0.86^f^	2.424 ± 0.286^c^
4	2.5	–	27.86 ± 1.33^h^	2.756 ± 0.304^a^
5	3.0	–	26.11 ± 1.42^h^	2.982 ± 0.477^a^
AC1	1.0	0.15	86.68 ± 3.22^c^	2.329 ± 0.121^c^
AC2	1.5	0.15	79.52 ± 2.87^e^	2.437 ± 0.117^c^
AC3	2.0	0.15	82.32 ± 1.66^d^	2.510 ± 0.122^b^
AC4	1.0	0.30	82.69 ± 1.23^d^	2.427 ± 0.183^c^
AC5	1.5	0.30	92.34 ± 2.43^b^	2.469 ± 0.155^b^
AC6	2.0	0.30	97.43 ± 1.77^a^	2.515 ± 0.158^b^
AC7	1.0	0.45	95.87 ± 2.12^a^	2.391 ± 0.266^c^
AC8	1.5	0.45	88.65 ± 0.91^bc^	2.496 ± 0.307^b^
AC9	2.0	0.45	78.38 ± 2.86^e^	2.616 ± 0.277^b^

Different letters a–g indicate significant differences (p < 0.05).

### Stability and release characteristics of phage microcapsules *in vitro* digestion

**FIGURE 5:**
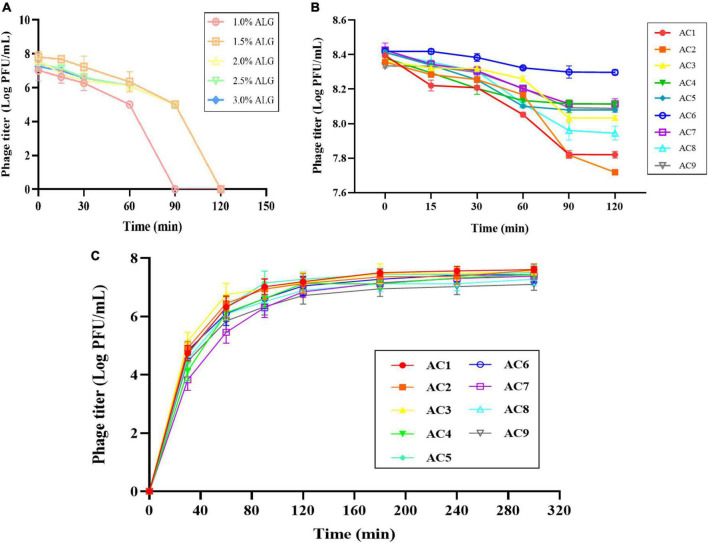
Stability and release characteristics of phage microcapsules *in vitro* digestion. The stability in SGF **(A)** Pure ALG microcapsules. **(B)** AC microcapsules. **(C)** The release behavior of phage loaded within AC microcapsules. AC1–AC9 represents polysaccharide mixtures in different proportions. (AC1) 1%ALG and 0.15%CG, (AC2) 1.5%ALG and 0.15%CG, (AC3) 2%ALG and 0.15%CG, (AC4) 1%ALG and 0.3%CG, (AC5) 1.5%ALG and 0.3%CG, (AC6) 2%ALG and 0.3%CG, (AC7) 1%ALG and 0.45%CG, (AC8) 1.5%ALG and 0.45%CG, (AC9) 2%ALG and 0.45%CG.

After preparing a solution of ALG microcapsules in SGF for 1 h, the phage titer was reduced by 1.7–2.9 log PFU/mL, and the phage was basically inactivated after 2 h (Figure 5A). When CG concentration was 0.15%, the phage titer decreased by 0.47 log PFU/mL, 0.69 log PFU/mL, and 0.33 log PFU/mL, respectively. When CG concentration was 0.30%, the phage titer decreased by 0.27 log PFU/mL, 0.33 log PFU/mL and 0.12 log PFU/mL, respectively. When CG concentration was 0.45%, the phage titer decreased by 0.31 log PFU/mL, 0.46 log PFU/mL, and 0.25 log PFU/mL, respectively (Figure 5B).

The release characteristics of phage-loaded microcapsules in SIF are shown in Figure 5C. Since the ALG microcapsule has a poor ability to protect phage in SGF, the titer of phage decreased significantly after 2 h in SGF, so the release of ALG microcapsule in SIF was not included in the results. In the first 30 min after entering SIF, microcapsules rapidly absorb water and swell, and the surface pores widen, resulting in massive release of phages in the early stage. In the following period, microcapsules can continue to release phages, and achieve almost 100% release at the end.

### Swelling properties of phage microcapsules during *in vitro* digestion

**FIGURE 6:**
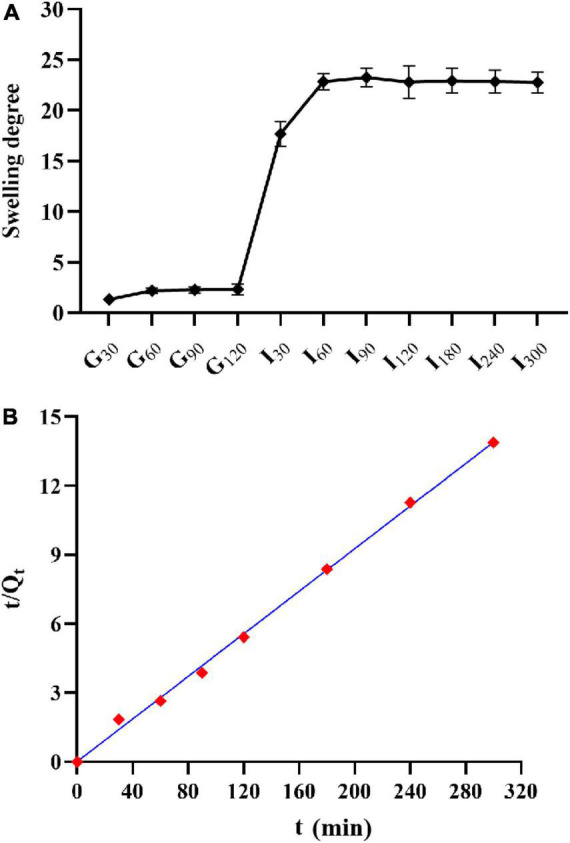
Swelling property of microcapsules. **(A)** Swelling degree and; **(B)** the relationship between the reciprocal t/Qt and t of the swelling degree of AC microcapsules.

The swelling degree of microcapsules in SGF reached about two times, but with the extension of time, the swelling degree did not increase (Figure 6A). After the microcapsules were transferred into SIF, the swelling degree of microcapsules could reach more than 20 times. In the first 30 min of SIF, the microcapsules could absorb water quickly, and the swelling phenomenon continued with the increase of time, but the rate slowed down.

### Lytic activity of non-encapsulated and encapsulated phages

**FIGURE 7:**
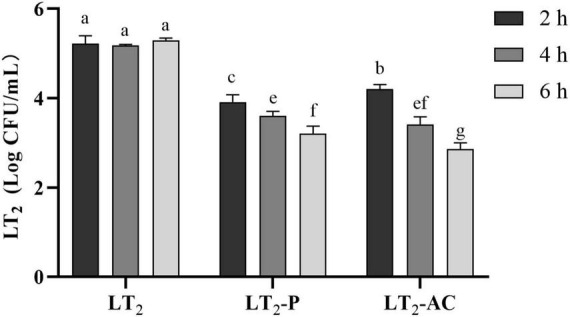
Reductions of *S. Typhimurium* LT_2_ were incubated with encapsulated and non-encapsulated phages in the gastrointestinal fluid at 37°C for 2, 4, and 6 h. (LT2) only was added *S. Typhimurium* LT_2_ as control, (LT_2_-P) added free phage SL01, (LT_2_-AC) added microencapsulated phage. Different letters a–g indicate significant differences (*p* < 0.05).

The lytic activities of non-capsulated and encapsulated phages were determined against LT_2_ over 2, 4, and 6 h in simulated intestinal conditions at MOI = 0.01 (Figure 7). Both free phage SL01 and encapsulated phage could lyse *Salmonella in vitro*, and the viable number of LT_2_ decreased by about 1 log CFU/mL. With the increase in lysis time, the number of lytic bacteria in the encapsulated phage group was higher, showing a persistent lytic ability compared to the free phage group.

## Discussion

The purpose of this study was to use ALG and CG microcapsules to encapsulate phages to ensure that the phages could pass through the extreme digestive environment, maintain their lytic activity, and be released when entering the intestinal digestion stage.

Temperature and pH are commonly considered as main factors influencing the shelf life of phages in the food environment (Bao et al., 2015; Wang et al., 2016). Phages are normally inactivated at high temperatures. However, it has been previously reported that some phages were able to survive under temperatures up to 60°C (Bao et al., 2015; Huang et al., 2018; Yang et al., 2020). The *Klebsiella pneumoniae* phage VTCCBPA43 isolated by Anand et al. (2020) can withstand a high temperature of 80°C. The *Bacillus cereus* phage PlyHSE3 isolated by Peng and Yuan (2018) was almost completely inactivated after incubation at 50°C for 1 h. The degree of tolerance to high temperature among different phages may be related to the structure of phages and the different types of host bacteria receptors affected by temperature (Rabiey et al., 2020). Previous studies also indicated that phages remained active at pH ranging from 4 to 10 (Bao et al., 2015). However, as reported by Shahin et al. (2019), phage VB-SDYS-ISF003 of *Shigella dysentery* could only maintain its activity between pH’s 7 and 9. Phage sensitivity to pH-value may be due to the changes in the spatial conformation of the receptor-binding proteins between phages and bacteria, the charge of the proteins in different pH environments (Yang et al., 2020). Due to the intolerance of phage to high temperature and some extreme environments, the encapsulation technology also avoids the selection of processing methods that may destroy the activity of phage. The extrusion method has become the first choice for the preparation method because of its simplicity, convenience, economy, and safety.

Experiments on the biological characteristics of phages have confirmed that phages are extremely easy to inactivate under extreme pH conditions (Figure 1). We compared the resistance of phages encapsulated using ALG microcapsules with ALG-CG (AC) microcapsules in a simulated gastric acid environment. It is evident that all concentrations of ALG microcapsules showed poor protection (Figure 5A). Moreover, different ratios of AC microcapsules also showed uneven protective ability (Figure 5B). Overall, the composite microcapsules greatly improved the phage viability in the gastric acid environment.

The encapsulation and loading efficiencies of core materials are crucial indexes when evaluating their efficiency as smart delivery systems. Maresca et al. (2016) produced nisin microcapsules using a 1.6% alginate solution and found microencapsulation efficiencies between 71 and 76%. In another study, the mean encapsulation efficiency was determined as 93.3% for phage macroencapsules produced with 2.2% ALG–chitosan mixture as the encapsulating material B (Ma et al., 2008). Śliwka et al. (2019) microencapsulated phage T4 in a 2% alginate-mannitol mixture solution and reported the mean encapsulation efficiency, which was determined as 98%.

In the present study, with the increase of ALG content, the encapsulation rate showed a trend of first increasing and then decreasing (Table 1). The results reveal that the amount of ALG content has a significant influence on the microencapsulation (*p* < 0.05). This can be explained by the fact that there is an increase in the number of biopolymer molecules per unit volume, which in turn increases the number of binding sites for the calcium ions (Narsaiah et al., 2014). ALG and CG have a high content of –COOH and –OH, respectively, and so a closely packed network could be formed due to crosslinking of the biopolymers by hydrogen bonds and ionic interactions. As a result, there was a decrease in the distance between the alginate and CG molecules, thereby promoting the formation of a more closely packed network structure (Yu et al., 2019).

The release behavior of phage encapsulated suggests that the release of the phage was mainly concentrated in the first 30 min of SIF (Figure 5C), e.g., when the microcapsules moved from the stomach to the small intestine. Under acidic gastric conditions, the network of cross-linked polysaccharides inside the microcapsules is more compact because of the reduction in electrostatic repulsion between the alginate chains, thereby leading to smaller pores (Abdelghany et al., 2017).

The swelling of hydrogel-type microcapsules can be divided into three main processes :(1) water molecules enter into the microcapsules; (2) the polymer chain relaxes inside the microcapsule; and (3) the polymer chain extends under the action of molecules and the microcapsule expands (Yu et al., 2019). The swelling degree of microcapsules in SGF reached about two times, but with the extension of time, the swelling degree did not increase. This may be due to microcapsules in the early to enter SGF part will absorb moisture, but alginate molecules on the -COO- began to gradually form the protonation -COOH, -COOH, and formed hydrogen bonds between the internal structure of the microcapsule become close, to prevent the water molecules to enter, swelling degree rise, no longer reach the equilibrium state (Díez-Peña et al., 2003). Microcapsules in this experiment reached equilibrium in SGF, which was similar to the results of Gu et al. (2021). With the increase of pH value, the hydrogen bond breaks and -COOH deprotonates into -COO-. The repulsion makes the microcapsule absorb water and expand rapidly. And the microspheres gradually become transparent, the surface becomes sticky, and the gel strength weakens.

Schott’s second-order swelling kinetic equation was used to evaluate the swelling process of microcapsules (Yılmaz and Pekcan, 1998). The formula is:


t/Qt=1/(KQ∞)2+t/Q∞

where, Q_∞_represents equilibrium swelling, Q_t_ represents swelling at t, and K represents a swelling rate constant. As shown in Figure 6B, t/Q_t_ has a good linear relationship with t, *R*^2^ > 0.99.

A controlled release can alter the kinetics of phage infection, delaying phage release and thus prolonging the time host bacteria are lysed. With the increase of time, bacteria can be lysed continuously, which greatly increases the bacteriostatic effect of phage.

## Conclusion

In conclusion, this study demonstrates that the phage core matrix material encapsulated by ALG/CG microcapsules s a protective effect on phage inactivation under low pH conditions and can maintain the release and lysis activity of phage over time. Microencapsulation represents a simple and inexpensive phage oral drug delivery system for zoonotic and pathogenic enteric colonization. These results can provide a new reference for the development of microencapsulated phage-based antimicrobial agents to ensure the quality and safety of animal food. In future studies, other phages will be encapsulated using the same embedding method and similar studies will be performed.

## Data availability statement

The original contributions presented in the study are included in the article/supplementary material, further inquiries can be directed to the corresponding author/s.

## Author contributions

YZ: conceptualization, data curation, and writing – original draft. DQ: conceptualization and writing – review and editing. DX: writing – review and editing. HY, WL, and JH: writing – review and editing. All authors contributed to the article and approved the submitted version.
